# Dignity and Abortion in Law, Philosophy, and Bioethics

**DOI:** 10.1017/jme.2025.10179

**Published:** 2026

**Authors:** Eric Scarffe

**Affiliations:** Florida International University, United States

**Keywords:** Dignity, Abortion, Philosophy, Law, LGBTQ+ Rights

## Abstract

Dignity has been a notoriously elusive concept to philosophers. Nevertheless, in the realms of politics, law, and policymaking, appeals to dignity are frequent, and do not always align with the understandings most commonly endorsed by the philosophical literature. This paper considers how “dignity” is frequently appealed to in ethical arguments about the permissibility of abortion, and argues that the judicial decisions related to reproductive and LGBTQ+ rights over the past 30 years in the United States offer deep insights into the nature of “dignity” that philosophers and other theorists ought to pay attention to. These insights not only have profound implications for our understanding of the nature of “dignity,” but also for ethical analysis more broadly.

Since the *Dobbs v. Jackson* decision was first leaked in the spring of 2022, there has been a resurgence of interest in abortion, both legally as well as ethically.^1^ This paper argues that while philosophers tend to presume an asymmetrical relationship between moral philosophy and the messy business of law and politics — with philosophy properly informing the practices of judges, policymakers, and politicians, but not vice versa — the recent debates surrounding abortion law demonstrate this presumption is misguided. There is plenty for moral philosophy to learn from the case law surrounding abortion, both as it relates to the ethics of reproductive rights as well as the nature of “dignity” itself.

This paper proceeds in three sections. The first section rehearses the standard ethical argument about abortion as it relates to concerns about dignity. Subsequently, the second section considers how the concept of “dignity” has been applied by the US Supreme Court in cases related to reproductive rights, but also more broadly. Ultimately, the last section argues that moral philosophers ought to learn from the way the court has increasingly recognized the importance of “dignitary harms” in its jurisprudence. Although the Court ultimately ignored its own precedent in *Dobbs*, there are two primary upshots from this analysis. First, it offers philosophers interested in the ethics of abortion, or the concept of “dignity” more generally, new directions with which to think about their work. Second, it calls to reorient philosophy’s relationship to practice: where practitioners and scholars in other fields do not merely *use* philosophical concepts, but actively participate in making and shaping them.

## I. Dignity in Abortion Ethics

The standard argument about reproductive rights and access to abortion, at least as it relates to ‘”dignity,” is well-trodden ground. Ronald Dworkin, for instance, characterized this position as the view that “abortion is wrong in principle because it disregards and insults the intrinsic value, the sacred character, of any stage or form of human life.”^2^ Indeed, I believe this argument can be fairly schematized in something like the following set of premises and conclusions:

- P1 Human dignity refers to the inviolable value of human beings (the value principle);
- P2 All human beings have dignity in equal measures (the equality principle);
- P3 Fetuses are human beings;
- C1 Therefore fetuses have dignity and inviolable value (P1 & P2);
- C2 Therefore abortion is impermissible.

In many respects, this position is more demanding than those that rely on claims about fetal personhood (e.g., Garland-Thomson & Reynolds,^3^ Milton^4^) or interests in their future life (e.g., Marquis,^5^ Ely^6^), but it is also more plausible as an argument. As Dworkin continues in the same passage, “[t]he belief that human life in any form has intrinsic, scared value can therefore provide a reason for people to object violently to abortion, to regard it as wicked in all circumstances, without in any way believing that a tiny collection of cells just implanted in the womb […] is already something with interests and rights.”^7^

Notably, this version of the argument also has the virtue of aligning with dominant views about abortion: both those who are predominantly against it, and those who are broadly in favor. For example, Pope John Paul II characterizes the Catholic Church’s position on the issue as follows: “[l]ike the first fratricide, every murder is a violation of the ‘spiritual’ kinship uniting mankind in one great family, in which all share the same fundamental good: equal personal dignity.”^8^ Indeed, Pope Paul II continues, it is the abandonment of “the inviolable dignity of the person” in contemporary life more broadly that can be blamed for many of societies’ social ills — as abandoning this value as the foundation for ethics and morals has not only resulted in “sinister” moral relativism, but also abandoning our basic commitments to one another as human beings.^9^

In a similar vein, something like this argument has been persuasive even to philosophers, such as Laurie Shrage, who are broadly supportive of reproductive rights. As Shrage writes, “[s]ome abortions, such as late nontherapeutic abortions, are inconsistent with any reasonable understanding of the principle that human life has inherent worth, and thus the government may restrict such abortions.”^10^ For Shrage, therefore, a commitment to P1 and P2 compels us to accept that, at some point, nontherapeutic abortions are inconsistent with the value principle or the equality principle. And while Shrage insists there should remain “broad therapeutic exceptions to abortion bans in the second trimester,”^11^ nevertheless proponents of reproductive rights should acknowledge that the policy frameworks established by *Roe v. Wade* (1973)^12^ and *Planned Parenthood v. Casey* (1992)^13^ fly in the face of this basic instinct. That is, having a commitment to the inherent worth and dignity of human beings requires us to permit more restrictions on abortion access earlier in a pregnancy than either *Roe* or *Casey* otherwise permitted.

## II. Legal Conceptions of Dignity

The section above rehearsed what I take to be the standard way “dignity” is appealed to in arguments surrounding abortion and reproductive rights. If we accept that human life has an inviolable value, independent of whatever capabilities that life exhibits, then there would seem to be at least a *prima facie* reason for thinking abortion is morally impermissible, and that democracies could choose to protect this value through legislation.

For some philosophers, as well as proponents of reproductive rights more broadly, this has led them to attempt to keep the terms of the abortion debate focused on *choice.* Indeed, it is now somewhat commonplace amongst philosophers to not only frame the abortion debate in terms of choice, but to define “dignity” itself as being substantially related to it. As James Griffin writes, “[w]hat we attach value to, what we regard as giving dignity to human life, is our capacity to choose and pursue our conception of a worthwhile life.”^14^ Like most Kantian conceptions of dignity, for Griffin, “dignity” is substantially related (and perhaps even derivative of) the capacity of human beings to act autonomously. As Griffin reiterates a little later in the same book: “autonomy is a major part of rational agency, and rational agency constitutes what moral philosophers have often called, with unnecessary obscurity, the “dignity” of the person.”^15^

Similarly, although Martha Nussbaum expresses concern with adopting an overly narrow conception of dignity derivative of capabilities such as rational capacity (which would seem to exclude some persons who suffer from severe mental disabilities), she maintains “that the best way to solve this complex problem is to say that full and equal human dignity is possessed by any child of human parents who has any of an open-ended disjunction of basic capabilities for major human life-activities.”^16^ As Dixon and Nussbaum later expand: “it would seem inconsistent if the [Capabilities Approach] refused all moral status to the fetus […] the [Capabilities Approach] does recognize that the fetus possesses a type of human dignity—although its dependent and merely potential status means that its type of dignity is distinctive, and not directly commensurable with that of independent human beings.”^17^

In short, for proponents of reproductive rights, one response has been to reimagine P1 and P2 to be more restrictive. Fetuses may not fail to have “interests” in a way that would give them *some* relevant moral status, but attributing “dignity” to them (at least in the early stages of pregnancy) is a misnomer. If we understand “dignity” to be a value derivative of the capacity for rational agency (like Griffin) or some other capability (like Dixon and Nussbaum), insofar as fetuses in these early stages of development fail to exhibit these human capabilities they should be excluded from inclusion within P1 and/or P2.

Ultimately, this paper will argue that this view is misguided. Human beings do not have dignity in virtue of their rational capacity or other capabilities; rather, we value this capacity, in part, *because* they have dignity. Perhaps more importantly for the purposes of this paper, however, I believe something like this view is closer to the way the Supreme Court in the United States has come to understand its use of this notoriously elusive concept. This section, therefore, takes up the way dignity has been used by the Court in cases related to abortion law, but also subsequent decisions (built off this rationale) related to the expansion of LGBTQ+ rights.

To begin, although *Casey* is often cited as the first time “dignity” is invoked in the Court’s jurisprudence related to abortion, this telling of the story ignores the long litigative history in the intervening years between *Roe* and *Casey.* Indeed, six years earlier, “dignity” appears to be first invoked in *Thornburgh v. American College of Obstetricians* (1986),^18^ where the Court considered whether a Pennsylvania law requiring “informed consent” violated the Court’s ruling in *Roe.* Writing for the majority, Justice Blackmun (the author of the majority opinion in *Roe*) now writes in *Thornburgh* that:

Our cases long have recognized that the constitution embodies a promise that a certain private sphere of individual liberty will be kept largely beyond the reach of government. That promise extends to women as well as to men. Few decisions are more personal and intimate, more properly private, or *more basic to individual dignity* and autonomy, than a woman’s decision -- with the guidance of her physician and within the limits specified in Roe -- whether to end her pregnancy.^19^

According to the Court, therefore, the Constitution protects a certain private sphere of individual liberty from government interference. And while it does not follow that any law or regulation that infringes upon an individual’s autonomy (e.g., seatbelt laws) is unconstitutional, those laws that seek to interfere with those basic personal and intimate decisions central to respecting individual dignity and autonomy are out of bounds.

Setting aside the politics of the potential for *Thornburgh* to have overturned *Roe* (with the decision narrowly being upheld five to four), one way to understand the final paragraphs in *Thornburgh* is as a response to the significant criticism *Roe* had faced in the intervening years for failing to center the woman in its decision.^20^ As Linda Greenhouse characterizes it, this final paragraph is not a revision of *Roe*, but it certainly “rephrased the rationale for *Roe* in language that was more directly centered on the woman than any of the Court’s previous formulations.”^21^ Indeed, the language of “dignity” similarly finds its way into subsequent decisions in the United States related to reproductive rights: making the woman, as well as her ability to make *choices* free from government interference, central to the landscape of abortion law and ethics for the next thirty years.

Nevertheless, by itself, the appeal to “dignity” found in *Thornburgh* leaves much to be desired. Although one could imagine an argument which justifies pride of place being given to respecting the dignity of women in reviewing legislation related to abortion, no such argument is found within the pages of the Court’s decision. The initial appeal to “dignity” made by the Court, therefore, would appear to engage with the standard argument rehearsed in Section I only implicitly. Perhaps the fetus has dignity, but (as the Court observes in *Thornburgh*) surely the woman does too. Even *if* the fetus has dignity, then, it does not follow that pregnant persons lack a right to make choices about whether to make sacrifices (related either to pregnancy or being a parent) free from government interference or compulsion. Thus, if we take commentators such as Greenhouse seriously in thinking that *Thornburgh* is centering the woman in the Court’s views on abortion, then it would seem to follow that the Court is (at least implicitly) committing itself to the view that respect for the dignity of the woman takes priority over that of the fetus.

In response to these sorts of ambiguities and reliance on implicit arguments, many philosophers may desire the Court to clarify its terms — for depending on what we understand “dignity” to mean, it might lead us to radically different conclusions. Article 1 of the German Basic Law (*Grundgesetz*), for instance, explicitly states both that “[h]uman dignity shall be inviolable” and that it is “the duty of all state authority” to respect and protect it. Subsequently, the German Federal Constitutional Court (GFCC) ruled in *Schwangerschaftsabbruch I* [Abortion I] (1975)^22^ that a 1974 reform to Germany’s criminal law, which decriminalized abortion without justification, violated this basic commitment. As the Court reasoned “the new law must do justice to the principle of the inviolability of nascent life, but, at the same time, strike a balance between the rights of the unborn child and the human dignity of a pregnant woman and her right to the free development of her personality. In this regard, the rights of one could not take absolute precedence over the rights of the other.”^23^ The GFCC, therefore, not only made explicit the argument that was implicit in *Thornburgh*, but in doing so it also reached the opposite conclusion. Namely, that insofar as human dignity is an “inviolable” value possessed by all human beings (including the unborn), then the state has an obligation to respect and protect this value even if it means curtailing the rights and choices of women under its jurisdiction.

Ultimately, I concur with Dixon and Nussbaum that, for better or worse, “[t]heories shape practical debates.”^24^ Indeed, philosophers of science (e.g., Thomas Kuhn,^25^ or Paul Feyerabend^26^) have long since argued that even basic empirical observations about the natural world are necessarily theory-laden — with our theories, biases, and assumptions shaping our observations of the world. Nevertheless, this paper argues that there are good reasons to resist the philosophical temptation for the Court to have committed itself to a theoretical understanding of “dignity” in advance, and the case law related to “dignity” developed through *Thornburgh* (1986), *Ohio v. Akron Center* (1990),^27^ *Casey* (1992), and *Lawrence v. Texas* (2003)^28^ represents a compelling example of how the kind of casuistic reasoning embodied by law can help us sharpen our understanding of contested concepts such as “dignity” (I will return to this point in more detail in Section III).

Turning now to *Akron* (1990), four years after *Thornburgh*, we again find the Court appealing to “dignity” in a case related to reproductive rights, only this time pulling in the opposite direction. Indeed, writing for the majority, in *Akron* Justice Kennedy concludes that while the decision to have an abortion “will embrace [the mother’s] own destiny and personal dignity” this is not to say any law restricting access to abortion in the second trimester would be unconstitutional.^29^ Indeed, in the case of the Ohio law requiring physicians to notify the parents in the event an unmarried, unemancipated, minor woman is seeking an abortion, Justice Kennedy found such a requirement to be constitutionally permissible. As Kennedy writes, “[i]t would deny all dignity to the family to say that the State cannot take this reasonable step in regulating its health professions to ensure that, in most cases, a young woman will receive guidance and understanding from a parent.”^30^ Thus, while the *dignity of the woman* secures the right to an abortion, according to Kennedy it is the *dignity of the family* that permits some restrictions on this right: at least in the context of women who are unmarried, or unemancipated, minors.

But what does any of this mean? First, one lesson from *Akron* is a reminder that not all understandings of dignity are of the Kantian variety—that is, not all understandings of dignity are related to the inviolable value of human beings. Indeed, as has been well-documented by philosophers such as Jeremy Waldron^31^ and Michael Rosen,^32^ historically speaking “dignity” was more commonly used to denote someone of particularly high rank or social status (e.g., nobility). As Waldron writes, “[i]n Roman usage, *dignitas* embodied the idea of the honor, the privileges, and the deference due to rank or office, perhaps also reflecting one’s distinction in holding that rank or office.”^33^ The point, in short, is that although Kantian-inspired understandings of “dignity” as substantially related to rational agency or other human capabilities is undoubtedly an important understanding of “dignity,” it is neither the only understanding available nor the understanding most frequently appealed to in American jurisprudence.^34^

The second lesson from *Akron* is to note that, despite the popular depiction of Lady Justice, in general judges don’t have scales, and the values at stake in a given case do not often lend themselves to “weighing” commensurate values or interests against each other. For Justice Kennedy and the majority of the Court in *Akron*, considering the dignity of the family (in addition to the dignity of the fetus) against the dignity of the mother did not result in the scales swinging wildly back in a direction more similar to the one adopted by Germany’s highest court; rather, the Court noted that a (previously unrecognized) dignity interest of the family should be taken into consideration, and holistically concludes that this may allow for some additional restrictions on abortions to be put in place in order to protect it.

It is against this backdrop that we finally arrive at *Casey* (1992), and, to the surprise of many Court watchers, Justice Kennedy joined the majority opinion upholding *Roe* (1973). In a plurality opinion — penned jointly by Justices O’Connor, Kennedy, and Souter — the opening line tells us that “[l]iberty finds no refuge in a jurisprudence of doubt” and concludes within a few initial paragraphs that “the essential holding of Roe v. Wade should be retained and once again reaffirmed.”^35^ Five pages later, then, we are told what the Court took the essential holding of *Roe* to be. As the Court writes,

Our law affords constitutional protection to personal decisions relating to marriage, procreation, contraception, family relationships, child rearing, and education. Our cases recognize ‘the right of the individual, married or single, to be free from unwarranted governmental intrusion into matters so fundamentally affecting a person as the decision whether to bear or beget a child’ […] These matters, involving the most intimate and personal choices a person may make in a lifetime, choices central to *personal dignity and autonomy*, are central to the liberty protected by the Fourteenth Amendment.^36^

It is perhaps only with the context of *Thornburgh* that observers can appreciate that the language of “dignity” was neither a novel invocation for cases related to reproductive rights, nor in the Court’s jurisprudence more generally.^37^ Further, against the backdrop of *Akron*, it should be apparent that the court is not simply failing to take into account other dignity-related considerations; rather, the Court is conscientiously affirming that it believes the dignity of the mother outweighs whatever other dignitary interest might be at stake (e.g., the dignity of the family or the dignity of the fetus).

In Section III, I will return to how these decisions may inform debates in moral philosophy about “dignity” and the moral permissibility of abortion. However, it is significant to note that the Court’s dignity-related jurisprudence did not end in *Casey*, but stood at the core of the expansion of LGBTQ+ rights over the next thirty years.^38^ Before returning to this important topic, therefore, it is worthwhile pushing onward to consider how appeals to “dignity,” and the Court’s understanding of the term, developed after *Casey.*

Turning now to *Lawrence* (a case which considered the constitutionality of a Texas law prohibiting sodomy), writing for the majority Justice Kennedy once again appeals to “dignity” as the guiding principle in this case. As Justice Kennedy writes:

This, as a general rule, should counsel against attempts by the State, or a court, to define the meaning of the relationship or to set its boundaries absent injury to a person or abuse of an institution the law protects. It suffices for us to acknowledge that adults may choose to enter upon this relationship in the confines of their homes and their own private lives and still retain their dignity as free persons.^39^

According to the Court, therefore, while there is a certain *dignity* marked by a person’s capacity to choose and act free from government interference, this sort of dignity does not require the government to provide limitless freedom to individuals.

Indeed, the government can rightly curtail many aspects of a person’s freedom and limit many *kinds* of choices one is permitted to make, without it rising to a level of degrading their dignity. For example, the government can rightly prohibit murder or public endangerment and can also impose penalties which curtail the freedom of those who violate these prohibitions, but the existence or enforcement of these laws does not degrade the dignity of persons under the jurisdiction of the state. In contrast, when the state makes laws which compel individuals to act only in those ways that are consistent with the state’s understanding of what is morally appropriate, the greater harm done to individual dignity is not the supposed moral transgression, but the compulsion of this behavior through the social and legal coercion of law.

To explicate more clearly, here is the language from *Lawrence* which puts this point most plainly:

The stigma this criminal statue imposes, moreover, is not trivial. The offense, to be sure, is but a class C misdemeanor, a minor offense in the Texas legal system. Still, it remains a criminal offense with all that imports for the *dignity of the persons* charged. The petitioners will bear on their record the history of their criminal convictions.^40^

According to the Court, therefore, the fact that laws such as the one in Texas were rarely enforced, or that the material penalties associated with them were relatively minor, was not pertinent to its determination as to whether such a law impugned the dignity of individuals. Instead, the Court reasoned that the fact that Texas had a law prohibiting sodomy inflicted a dignitary harm on same-sex couples insofar as it criminalized activity understood to be within that sphere of liberty that is properly beyond the reach of government interference. In short, the Court recognized that *criminalizing* certain actions may not only pose a threat to the liberty of certain persons who violate the state’s edicts, but it also undermines the *dignity* of those under its dominion by coercing their otherwise free choices. This is not to say that a government can never be justified in curtailing a person’s freedom or coercing their decision-making, or that it is a person’s *capacity* to engage in this sort of decision-making that gives them dignity. Rather, the decision in *Lawrence* (2003) notes that there exists at least a thumb on the scale against the presumption of the validity of laws that curtail or prohibit individual freedom or choices, and that considering the implications the proposed legislation has for the *dignity* of individuals is significant to determining the law’s constitutionality.

Of course, *Lawrence* is not the only place we find this sort of analysis. Indeed, more than a decade later, Justice Kennedy reaffirms this view in *Obergefell v. Hodges* (2015)^41^: where the Court found there to be a constitutionally protected right not to be excluded from the marriage right. As Justice Kennedy notes, “excluding same-sex couples from the marriage right imposes stigma and injury of the kind prohibited by our basic charter.”^42^ These injuries, Kennedy resolves, are the sort of “dignitary wounds” previously described in *Lawrence*, and, as such, led the Court to find in favor of same-sex couples seeking to invalidate the Ohio law which prohibited same-sex marriage.^43^ As Kennedy concludes the Court’s majority decision, “[t]hey ask for equal dignity in the eyes of the law. The Constitution grants them that right.”^44^

One takeaway from this analysis is that *Dobbs* did more than overturn a federally protected right to an abortion — it undermined the entire foundation upon which LGBTQ+ rights were premised. Although appeals to “dignity” are widespread in the Court’s jurisprudence on issues ranging from criminal procedure,^45^ to the death penalty,^46^ states’ rights,^47^ and foreign sovereign immunity,^48^ the string of cases which supported the expansion of LGBTQ+ rights (including *Lawrence*, United *States v. Windsor* (2013),^49^ and *Obergefell*) were deeply intertwined with the Court’s jurisprudence on reproductive rights.^50^

The other takeaway, that has received less attention in the months and years after *Dobbs*, is to note the way in which the Court’s rulings in these cases did not merely *use* the language of dignity imported from philosophy, they reimagined it. As I have argued elsewhere, Kennedy’s use of dignity does not draw on a singular philosophical conception (e.g., Kantian-dignity) or previous usage by the court (e.g., the dignity of foreign sovereigns); rather, Kennedy’s use of dignity weaves together these seemingly disparate conceptions in novel ways that help illuminate this otherwise elusive concept.^51^ Part of what we see in this string of cases, therefore, is the Court developing an understanding of dignity that both recognizes the plurality of dignity interests at stake in a given case (e.g., the dignity of the mother, the fetus, and the family), as well as one that recognizes how legislation curtailing individual choices or criminalizing behavior may have significant import for the dignity of individuals. That is, the act of criminalizing certain behavior harms the dignity of individuals by treating them *as if* they are criminals worthy of condemnation, “with all that imports for the *dignity of the persons* charged.”^52^ Thus, in the same way the acknowledgement of the dignity of the family in *Akron* resulted in the Court revising its doctrine to permit *more* restrictions on abortions in the second trimester, its decision in *Lawrence* (and in particular its recognition of *dignitary harms*) should have led the Court to recognize stronger, not weaker, protections from government interference for decisions related to abortion procedures.

## III. Lessons for Bioethics and Moral Philosophy

But what can any of this tell us about the moral case? As John Austin famously noted, “[t]he existence of law is one thing; its merit or demerit is another.”^53^ In short, the mere fact that something is deemed legal or illegal does not logically entail anything about its morality (or vice versa). Indeed, the history of legal positivism has long held that there is no necessary relationship between law and morality, and therefore philosophers, legal practitioners, and laymen alike would do well not to confuse the two issues.^54^

This paper does not purport to undermine the validity of legal positivism’s separation thesis, although I believe one should resist reading more into it than many have assumed.^55^ Nevertheless, the first lesson the legal cases surrounding abortion law in the United States highlights for *moral* arguments about abortion is the competing dignity interests at stake. That is, even if we grant (as Shrage appears to do^56^) that at some point the fetus has *dignity*, this does not settle the question of what we should do: either personally or as a matter of public policy. Indeed, the mother’s dignity seems to be most present (and important) in considering how she thinks about her body, the risk(s) involved in pregnancy, and the prospect of having a child or being a parent. Thus, even if we grant that a fetus has human dignity in the relevant sense (either from the point of conception or at some later point), it is not clear that the standard argument is sufficient for warranting its conclusion that abortion is immoral. In short, there exists a logical gap between the conclusion that fetuses have dignity and inviolable value (C1) and the conclusion that abortion is morally impermissible (C2) — as there exist countervailing reasons and values that can lead one to the opposite conclusion.

Further, the case only appears to get stronger when we move into the realm of public policy. Indeed, relevant to public policy discussions is not only considerations of individual rights and moral values, but also what law communicates. As Catherine MacKinnon notes, “law is a particularly potent source and badge of legitimacy, and site and cloak of force.”^57^ When states make laws prohibiting or restricting abortion, then, this is not merely one policy decision amongst many possible alternatives; rather, it communicates to women and pregnant persons who might be seeking or considering an abortion that there is something wrong with this decision — and this inflicts a dignitary wound upon these persons. A wound that, as Justice Kennedy notes in *Obergefell*, “cannot always be healed with the stroke of a pen.”^58^

The second lesson for the moral case generalizes more broadly. Namely, talking about dignity as an inviolable value is not particularly helpful in determining what you should do, at least not if our modes of moral reasoning do not adequately adjust to accommodate this sort of input. Yes, the point is well taken by Kant that the dignity of humanity is without price;^59^ however, if we use this as a bludgeon to protest any decision that infringes upon, or risks, this value, something has gone significantly awry. Indeed, it seems to me that this reveals a problem with utilitarian-style ethical argument more broadly. That is, once an infinite value is placed upon a utilitarian scale, there is nothing that can bring the scales back into balance. We see a similar problem, for example, in discussions surrounding the relative importance of x-risks in effective altruism.^60^ What follows from this isn’t that we should abandon talk of non-fungible, inviolable values in ethics, nor does it suggest we should abandon any consideration of utility or cost-benefit analysis. Rather, moral philosophers must accept that referencing an inviolable, or infinite, value such as human dignity is not sufficient to settle the issue — not just because public policy decisions *will* consider more than these values, but because more values and considerations are relevant to even a purely moral analysis.

Further, if we are to accept that talk of inviolable values is important for discussions in applied ethics or other fields of moral/political discourse (which I believe they are), then it would follow that political/moral philosophers would do well to adopt language and modes of reasoning that are better suited to sorting through the competing interests and values at stake. This could include adopting casuistic forms of reasoning grounded in practices more similar to those found in the common law: reasoning from one case to the next by analogy through a careful articulation of the similarities and differences.

Adopting such a methodology may also better position the abortion debate to allow different perspectives to be in active conversation with each other. Although I believe Paltrow et al. are correct in their assessment that the criminalization of abortion is “is rooted in intersecting policies and ideologies that have both racism and sexism at their core,” this has triggered significant backlash from earnest anti-abortion proponents who feel their argument is ignored by such analysis.^61^ As Smith argues, to “accuse pro-lifers of racism because African American women have a large percentage of abortions and would be disproportionately impacted by access restrictions” seems fundamentally misplaced.^62^ Rather than talking past one another, a shift in methodology may allow these positions to come into conversation: either by having the conversation directly on the dignity-based grounds scholars such as Smith seem to demand, or by allowing reasons such as those advanced by Paltrow et al. to factor into a holistic analysis inspired by casuistry and the common law.

This brings me to my last point, namely: the above series of cases help to demonstrate why the presumed hierarchy (or, as Elizabeth Anderson puts it more charitably, “division of labor”^63^) between philosophy and practitioners is misguided. As Kristen Hessler has convincingly argued, while it is surely among the virtues of moral philosophy to be free from some of the formal and/or political constraints of reasoning in law and politics, this is not an unqualified good — for without similar pressures to engage with novel issues, or historically marginalized stakeholders, moral theorists risk ignoring deeply entrenched biases and assumptions in their analysis. According to Hessler, traditional, and largely abstract, moral theorizing must therefore recognize that its methodology may predispose it to a unique “epistemic obstacle” that other disciplines and institutions may be better situated to overcome.^64^

For example, while there exist many social and cultural barriers to bringing a case to court, the motivation to bring a case to court is significantly higher than any corresponding motivation to bring a case to the attention of moral philosophers. As such, even if the demographics within professional philosophy were similar to those found in the legal profession, the kinds of stakeholders and cases the legal profession engage with have built-in pressure to be more representative of the population’s experiences. This is no guarantee that this leads to better reasoning or better outcomes, but it does create a forum in which baked-in assumptions can get challenged and the experiences of marginalized voices heard.

For these reasons, I think carefully considering the way the conversations about “dignity,” and its relationship to reproductive rights (and individual rights more broadly), have played out in the legal sphere may be more productive to philosophers than some might otherwise presume. Indeed, in recent years there has been no shortage of debate amongst philosophers about what, if anything, dignity means, and whether it continues to have any use whatsoever in our moral vocabularies (e.g., Killmister,^65^ Etinson,^66^ Sangiovanni,^67^ Rosen^68^). Without a doubt, I believe many of these philosophical debates could positively contribute to the *legal* understandings of dignity appealed to by courts. However, I believe the inverse to also be true. “Dignitary harms” are not merely a quaint part of the history of sumptuary laws, or the harm associated with insulting the king; rather, dignitary harms are invariably tied up in thinking about what law communicates when it prohibits things like abortion, and what harm might be done to individuals as a result.

## IV. Conclusion

This paper argues that the standard argument about dignity and abortion suppresses several important considerations. This includes, of course, competing dignity interests at stake in decisions about whether to continue with a pregnancy (e.g., the dignity of the mother), but also the *dignitary harm* inflicted upon women and pregnant persons when a state prohibits access to abortion services under law. For politicians, policymakers, and judges, I believe this should cause them either to rethink their decision in *Dobbs*, or at the very least influence the way they think about legislation related to abortion and reproductive rights more carefully at the local, state, and federal levels.

More generally, however, this paper has also argued that this example should encourage a reorientation in philosophy. Philosophers do not have privileged access to a platonic realm of forms. This is not to say philosophers have nothing to contribute to other disciplines whatsoever; rather, we must more forcefully reject the assumption that the relationship between philosophy and other disciplines is unidirectional. Philosophers do not bring “truths” back down from on high to be used by practitioners and other fields of study; rather, any “truths” arrived at must pay careful attention to the actual cases (and people) such “truths” are intended to apply to and be about.
